# The propionate-GPR41 axis in infancy protects from subsequent bronchial asthma onset

**DOI:** 10.1080/19490976.2023.2206507

**Published:** 2023-05-02

**Authors:** Takashi Ito, Yumiko Nakanishi, Ryohei Shibata, Noriko Sato, Toshi Jinnohara, Sayo Suzuki, Wataru Suda, Masahira Hattori, Ikuo Kimura, Taiji Nakano, Fumiya Yamaide, Naoki Shimojo, Hiroshi Ohno

**Affiliations:** a Laboratory for Intestinal Ecosystem, RIKEN Center for Integrative Medical Sciences, Yokohama, Japan; b Department of Pediatric Surgery, Graduate School of Medicine, Chiba University, Chiba, Japan; c Department of Pediatrics, Graduate School of Medicine, Chiba University, Chiba, Japan; d Laboratory for Microbiome Sciences, RIKEN Center for Integrative Medical Sciences, Yokohama, Japan; e Department of Signal Transductions, Graduate School of Biostudies, Kyoto University, Kyoto, Japan; f Department of Applied Biological Science, Graduate School of Agriculture, Tokyo University of Agriculture and Technology, Tokyo, Japan; g Center for Preventive Medical Sciences, Chiba University, Chiba, Japan; h Laboratory for Immune Regulation, Graduate School of Medical and Pharmaceutical Sciences, Chiba University, Chiba, Japan; i Immunobiology Laboratory, Department of Medical Life Science, Graduate School of Medical Life Science, Yokohama City University, Yokohama, Japan

**Keywords:** Bronchial asthma, allergic disease, microbiota, Scfas, propionate, lactation period, GPR41, Tlrs, human birth cohort

## Abstract

Evidence has accumulated that gut microbiota and its metabolites, in particular the short-chain fatty acid propionate, are significant contributors to the pathogenesis of a variety of diseases. However, little is known regarding its impact on pediatric bronchial asthma, one of the most common allergic diseases in childhood. This study aimed to elucidate whether, and if so how, intestinal propionate during lactation is involved in the development of bronchial asthma. We found that propionate intake through breast milk during the lactation period resulted in a significant reduction of airway inflammation in the offspring in a murine house dust mite-induced asthma model. Moreover, GPR41 was the propionate receptor involved in suppressing this asthmatic phenotype, likely through the upregulation of Toll-like receptors. In translational studies in a human birth cohort, we found that fecal propionate was decreased one month after birth in the group that later developed bronchial asthma. These findings indicate an important role for propionate in regulating immune function to prevent the pathogenesis of bronchial asthma in childhood.

## Introduction

Bronchial asthma (BA), one of the most common allergic diseases in routine clinical practice, is an allergic airway inflammatory disease in which there is an adverse reaction to foreign antigens such as dust mites, pet hair and fungi^1,2^. BA is divided into two subtypes. Atopic asthma is more common and involves allergic reactions to common environmental allergens with elevated levels of IgE in the blood. The other is non-atopic asthma, which is relatively rare, and allergens are not involved; instead obesity and fragile airways are likely implicated^3^. It is estimated that more than 300 million people suffer from BA worldwide and there is an urgent need from a public health perspective to understand the pathogenesis of BA to contain the disease^1,2^.

Maternal lifestyle during the prenatal and lactation periods is known to be an important determinant for the establishment of gut microbiota and the development of BA in their children^4^. Large human epidemiological studies suggest an association between the onset of BA and childhood circumstances, such as the method of infant feeding^5^, the method of delivery^6^ and the presence of pet animals^7^.

In addition, gut microbiota has been implicated in bronchial asthma, especially pediatric bronchial asthma. Gut microbiota changes most dramatically after birth and during infancy and is influenced by the mode of delivery, nutrition, and the maternal skin and gut microbiota. In addition, there are reports showing that changes in gut microbiota during infancy are involved in allergic diseases, particularly bronchial asthma.　For example, Arrietta et al. reported that *Lachnospira*, *Veillonella*, *Fecalibacterium*, and *Rothia* were decreased, accompanied by reduced acetate, in the feces at 100 days after birth in infants at risk of asthma at one year of age^8^.　Moreover, when these authors transplanted feces from an infant at risk of asthma into GF mice, OVA-induced allergic airway inflammation was exacerbated; the allergic airway inflammation was ameliorated by the addition of the above four bacteria to the feces from the same infant^8^. On the other hand, another study reported that the diversity of gut bacteria at one and 12 months correlated with serum IgE and allergic conjunctivitis but not with the onset of bronchial asthma^9^, suggesting the need for further investigations to understand unambiguously the relationship between the gut environment and pediatric asthma.

In recent years, metabolomic analysis has attracted much attention, especially in the intestinal tract, where short-chain fatty acids (SCFAs) and other microbial metabolites affect host immune responses that maintain intestinal homeostasis, and whose dysregulation can lead to disease^10^. In particular, propionate is a major microbial fermentation metabolite in the gut of many animals including humans, and exerts versatile health-promoting effects systemically beyond the gut^11^, including the attenuation of cardiac hypertrophy, fibrosis and vascular dysfunction^12^, as well as the amelioration of colonic inflammation^13^. These studies suggest that propionate in the gut could modulate the pathogenesis of systemic diseases and gut-specific immune responses. However, little is known about whether intestinal propionate is involved in allergic diseases such as BA.

In the present study, we performed mouse studies to show that propionate intake through breast milk during the lactation period can ameliorate airway inflammation in the offspring in a house dust mite-induced asthma model by suppressing eosinophil function through GPR41 signaling. Moreover, a human birth cohort study with fecal microbiome and water-soluble metabolome analyses suggested that decreased fecal propionate in the first month of life was associate with subsequent BA development.

## Results

### Propionate suppresses the asthmatic phenotype

**Figure 1.**
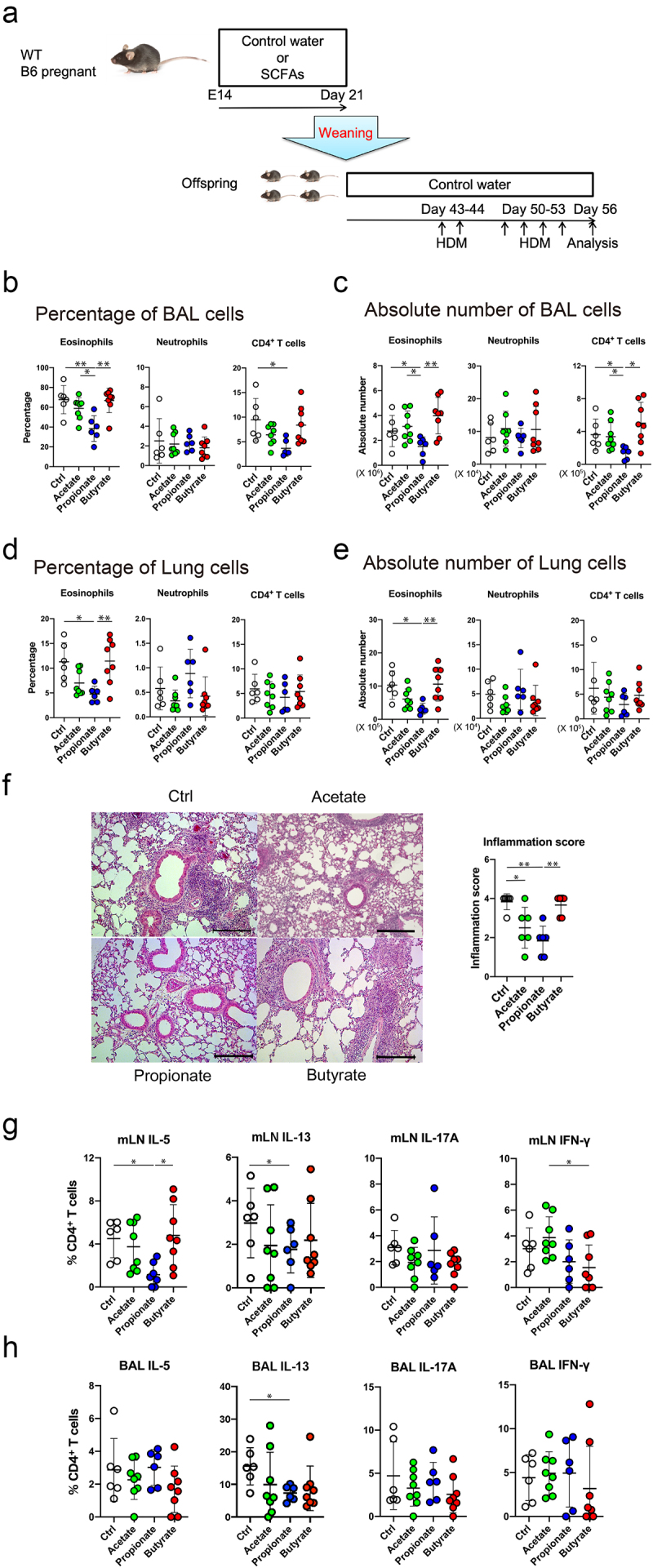
Propionate intake attenuates the eosinophilic airway inflammation and Th2 cytokine production in a murine HDM-induced asthma model. (a) Schematic depiction of the experimental protocol. Details are described in the methods section. (b-e) the percentage (b, d) and absolute number (c, e) of inflammatory cells in the bronchoalveolar lavage (b, c) and lung (d, e) were evaluated 72 h after the last HDM challenge. Data are mean ± SD. *p < 0.05 and **p < 0.01 by one-way ANOVA and Tukey’s test. (f) Representative photomicrographs of lung sections with hematoxylin and eosin staining and histological inflammatory scores. Bars = 50 µm. Data are mean ± SD. *, *p < 0.05 and **p < 0.01 by one-way ANOVA and Tukey’s test. (g, h) the percentage of IL-5-, IL-13, IL-17A, or IFN-γ-producing CD4+ cells in the mediastinal lymph node (mLN) (g) and bronchoalveolar lavage (BAL) (h) by flow cytometry intracellular staining analysis. Data are mean ± SD. *p < 0.05 by one-way ANOVA and Tukey’s test.

To ask whether propionate intake during the lactation period could attenuate allergic airway inflammation later in life, we used a mouse model of allergic airway inflammation by intratracheal administration of house dust mite (HDM). To this end, newborn mice ingested propionate or other SCFAs during the lactation period through the breast milk of their mothers, who had *ad libitum* access to drinking water containing acetate, propionate or butyrate, or control plain water (Figure 1a). The offspring were then given plain water after weaning at 3 weeks of age and at 6 weeks of age they were sensitized and challenged by intratracheal administration of HDM extract. The numbers of eosinophils, neutrophils and CD4^+^ T cells in the BALF and lung were then measured (Figure 1a). Among the groups, propionate-fed mice had fewer eosinophil and less CD4+ T cell exudation in the airways compared with mice in the other groups (Figure 1b,c). The infiltration of eosinophils in the lung was also significantly reduced in the propionate-exposed mice (Figure 1d,e). These results were also corroborated in mice in which SCFA administration was started immediately after birth (Supplementary Fig. S1C-F).　Moreover, to determine whether these differences in eosinophil counts were due to sex differences, we performed the same experiment in male mice and found that propionate administration decreased eosinophil counts in them as well (Supplementary Fig. S1G-J). Histological analyses also revealed a significant decrease in eosinophil and neutrophil infiltration around the bronchi in the propionate group compared to the other groups of mice (Figure 1f). These results suggest that the administration of propionate during the lactation period attenuated subsequent allergic airway inflammation in mice. Furthermore, HDM-induced production of Th2 cytokines interleukin (IL)-5 and IL-13 by mediastinal lymph node (mLN) cells and bronchoalveolar lavage (BAL) cells was significantly decreased in the propionate group compared with the other three groups; by contrast, a Th17 cytokine IL-17A and a Th1 cytokine IFN-γ were comparable among all four groups (Figure 1g,h). Collectively, these results indicate that the increased intestinal propionate intake in early infancy by administration of propionate during lactation attenuates the development of HDM-induced Th2 responses and thus suppresses the allergic phenotype later in life.

### GPR41 mediates the suppressive effect of propionate on airway allergy

**Figure 2.**
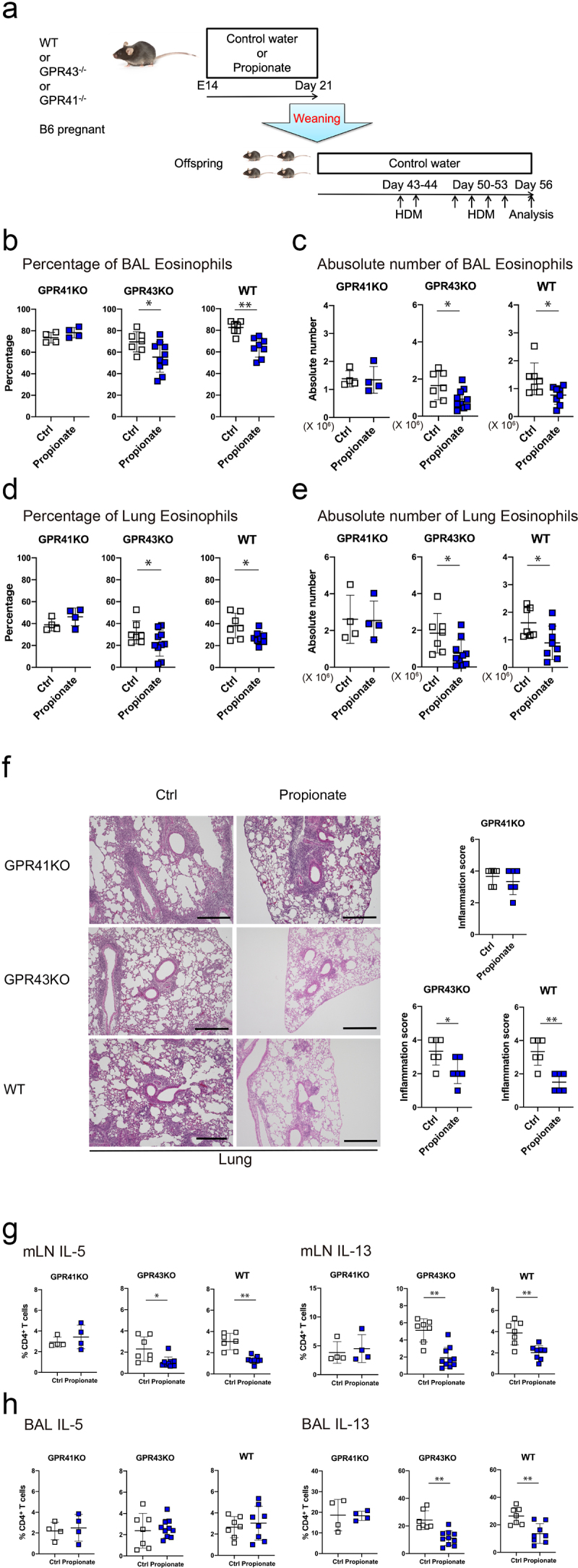
GPR41 deficiency eliminates the inhibitory effect of oral propionate administration in a murine HDM-induced asthma model. (a) Schematic depiction of the experimental protocol. Details are described in the methods section. (b-e) the percentage (b, d) and absolute number (c, e) of eosinophils in the bronchoalveolar lavage (b, d) and lung (c, e) were evaluated 72 h after the last HDM challenge. Data are mean ± SD. *p < 0.05 and **p < 0.01 by student’s t-test. (f) Representative photomicrographs of lung sections with hematoxylin and eosin staining and histological inflammatory scores. Bars = 50 µm. Data are mean ± SD. *, *p < 0.05 and **p < 0.01 by Student’s t-test. (g, h) the percentage of IFN-γ-, IL-4-, IL-5-, IL-13, or IL-17A-producing CD4^+^ cells in the mediastinal lymph node (mLN) (g) and bronchoalveolar lavage (BAL) (h) cells by flow cytometry intracellular staining analysis. Data are mean ± SD. *p < 0.05 by student’s t-test.

It is well known that SCFAs, including propionate, exert some of their effects via G protein-coupled receptors (GPCRs) GPR41 and GPR43 expressed by gut epithelium and immune cells, which play a crucial role in maintaining homeostasis in the gut^14^. Accordingly, we analyzed the involvement of GPR41 and GPR43 in the propionate-mediated suppression of the development of HDM-induced allergic airway inflammation. WT, GPR41 knockout (GPR41^−/−^) and GPR43 knockout (GPR43^−/−^) pregnant mice were divided into a propionate water-drinking group and a control water-drinking group. Propionate was added to the drinking water during the 3 weeks of lactation and the offspring mice were sensitized and challenged with HDM by intra-tracheal administration (Figure 2a). The number of inflammatory cells and cytokine production by the lung and BAL cells were then evaluated. Notably, GPR41^−/−^ mice showed comparable numbers of eosinophils and CD4+ T cells in the BALF and lung-infiltrating eosinophils and neutrophils regardless of propionate exposure (Figure 2b-e, Supplementary Figure S2A-D). Moreover, GPR41 deficiency eliminated the propionate-induced suppression of peribronchial and perivascular inflammation (Figure 2f). The reduced HDM-specific IgG1 production induced by propionate treatment was also abolished in GPR41^−/−^ mice (Supplementary Figure S2E). Furthermore, the production of IL-5 and IL-13 by both mLN cells and BAL cells was comparable between the propionate exposed and control GPR41^−/−^ mice (Figure 2g,h). By contrast, in GPR43-knockout mice, as with WT mice, propionate still ameliorated the allergic airway inflammation and suppressed Th2 cytokine production (Figure 2a-d,g,h). These data suggest that GPR41, but not GPR43, acts as a propionate receptor to protect from HDM-induced allergic airway inflammation in mice.

**Figure 3.**
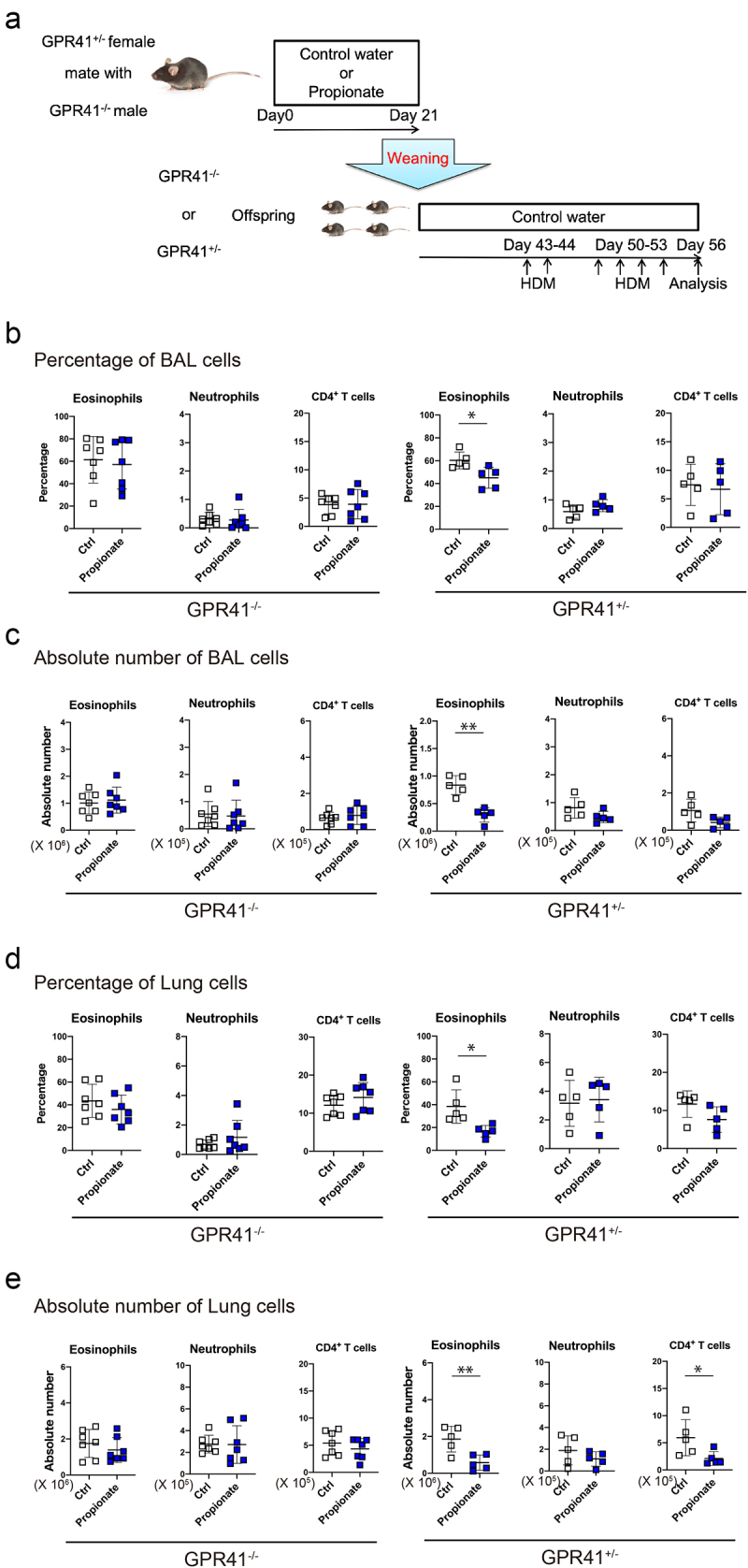
The inhibitory effect of propionate was eliminated despite GPR41 expression by the mother.(a) Schematic depiction of the experimental protocol. (b-e) the percentage (b, d) and absolute number (c, e) of neutrophils and CD4^+^ T cells in the bronchoalveolar lavage (b, d) and lung (c, e) were evaluated 72 h after the last HDM challenge. Data are mean ± SD. *p < 0.05 and **p < 0.01 by student’s t-test. .

To distinguish whether GPR41 expression in the mother or offspring mice is involved in propionate-induced suppression of HDM-induced allergic airway inflammation, we crossed GPR41-heterozygous females with GPR41-homozygous knockout males, and the resulting GPR41-heterozygous and GPR41-homozygous knockout offspring were used (Figure 3a). We still observed the suppressive effect of propionate on the airway inflammation in GPR41-heterozygous offspring, which was eliminated in GPR41-homozygous knockout offspring (Figure 3b-e), suggesting that this propionate effect depends on GPR41 expression in the offspring themselves and not in the mothers.

### TLR expression is upregulated in eosinophils from propionate-treated mice

**Figure 4.**
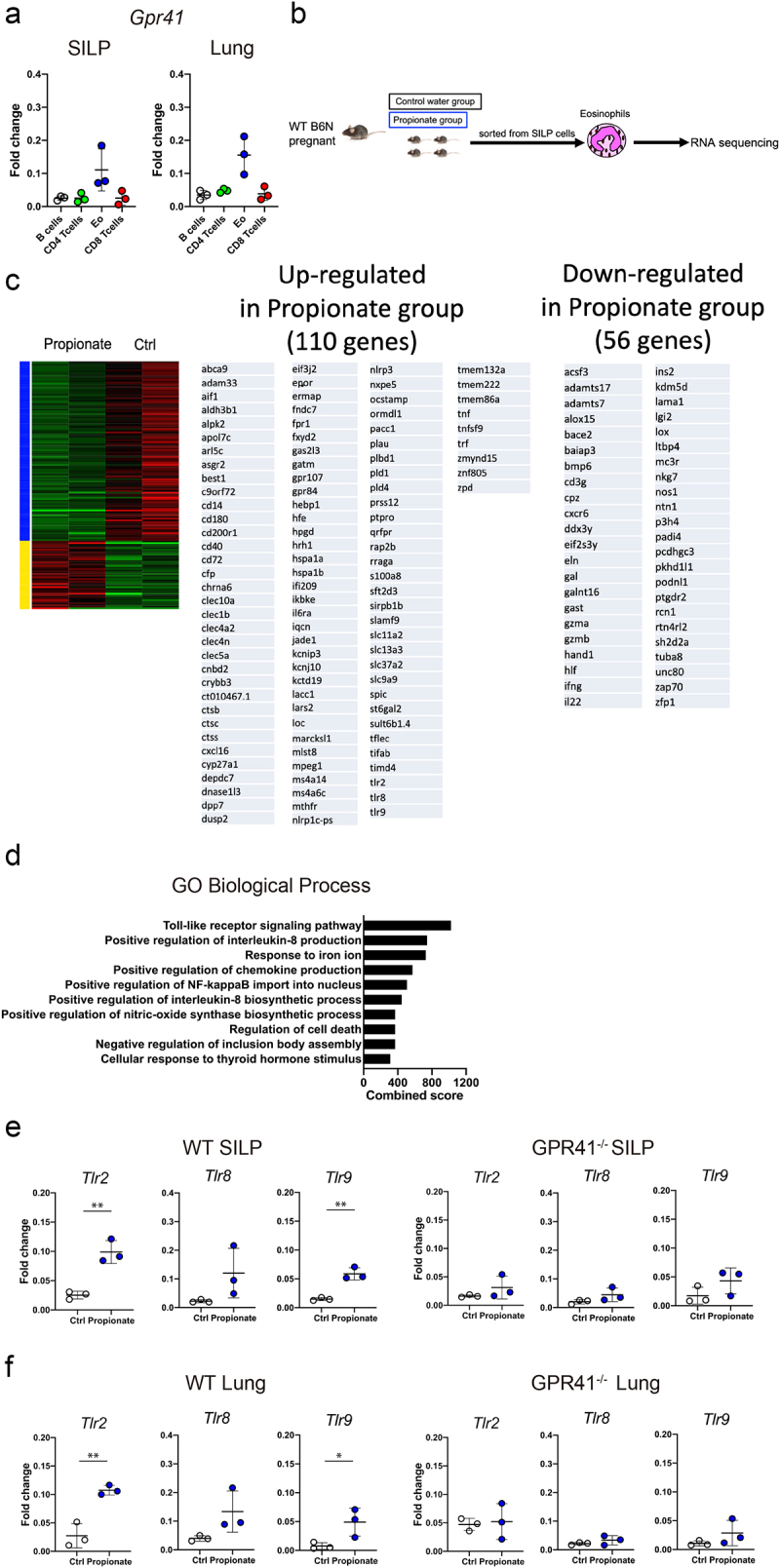
TLR family genes are upregulated in intestinal eosinophils of offspring born from mice fed with propionate (a) Expression levels of Gpr41 mRNA were evaluated by qPCR in eosinophils, B cells, CD4^+^ T cells, and CD8^+^ T cells isolated by FACS from small intestinal lamina propria (SILP) and lung cells of wild-type C57BL/6 mice. (b) Schematic depiction of the experimental protocols for isolation of mRnas used in (c-e). (c) Heatmap of differentially expressed genes (DEGs) between the Propionate group and Control water group and a list of the genes upregulated (middle) and downregulated (right) in the Propionate group. (d) Bar plots of the combined score for gene ontology (GO) term enrichment in GO biological processes for DEGs between the propionate group and the Control water group. (e) Expression levels of Tlr2, Tlr8 and Trl9 mRNA in wild-type and GPR41 knockout SILP eosinophils were evaluated by qPCR. Data are mean ± SD. **p < 0.01 by Student’s t-test. (f) Expression levels of Tlr2, Tlr8 and Trl9 mRNA in lung eosinophils were evaluated by qPCR. Data are mean ± SD. *p < 0.05 and **p < 0.01 by Student’s t-test.

To identify the cells in the intestine expressing GRP41, we examined *Gpr41* expression by qPCR analysis of cells sorted from the small intestinal lamina propria (SILP) and lungs of wild-type mice. Interestingly, *Gpr41* was strongly expressed in eosinophils, from both SILP and lungs, among the various immune cells examined (Figure 4a).　This result suggests that GPR41-expressing eosinophils are likely to be the main target of the ingested propionate.

In order to perform a bias-free and comprehensive genetic screening of intestinal eosinophils, we next performed RNA sequencing analysis on SILP eosinophils sorted from the offspring of mothers fed with propionate-containing or control water (Figure 4b). One hundred ten genes were upregulated and 56 genes were downregulated in the eosinophils from propionate-fed mice (Figure 4c). Surprisingly, Gene Ontology (GO) revealed that, among the genes upregulated in the propionate-fed group, genes belonging to the toll-like receptor (TLR) signaling pathway in GO biological processes (GO0002224) were the top hit in the combined score ranking (Figure 4d). Consistent with this observation, mRNAs encoding TLR2, TLR8 and TLR9 were highly expressed in SILP eosinophils isolated from the propionate-treated mice compared to the control mice (Figure 4e). By contrast, propionate treatment did not enhance TLR family expression in GPR41 knockout mice (Figure 4e). Finally, analysis of the TLR family in lung eosinophils showed that its expression was also increased in propionate -treated mice (Figure 4f). These results suggest that propionate may facilitate TLR signaling in intestinal eosinophils and also affect lung eosinophils through GPR41.

### Propionate is decreased in asthma patients

Finally, we performed fecal metabolome and microbiome analyses on a human birth cohort to address the contribution of propionate in infants to the subsequent development of BA in humans.　In this birth cohort, 269 pregnant women, who either themselves and/or the babies’ fathers had a history of allergic diseases, were recruited and agreed to participate^15^. Fecal samples were collected from these babies/children at one week, one month, one year, and five years of age and breast milk samples were collected from mothers at one week, one month, and six months of ages. At five years of age, 65 patients were unable to continue with the sample collection due to withdrawal of consent or change of hospital. Finally, fecal and breast milk samples from 204 participants were then collected. The participants were classified into two groups based on clinical diagnosis by pediatricians: the BA-onset group (BA group, *n* = 23) and the non-BA group (NBA group, *n* = 181) (Supplementary Fig. S3A). When we analyzed the clinical data for the BA and NBA groups, we found a higher proportion of boys, as well as a higher incidence of other allergic diseases such as food allergy and atopic dermatitis, in the BA group compared to the NBA group (Supplementary Fig. S3B), which is consistent with existing clinical studies on asthma in children^16–18^. On the other hand, there was no difference in gestation period or frequency of antibiotic use between the BA and NBA groups (Supplementary Fig. S3B).

**Figure 5.**
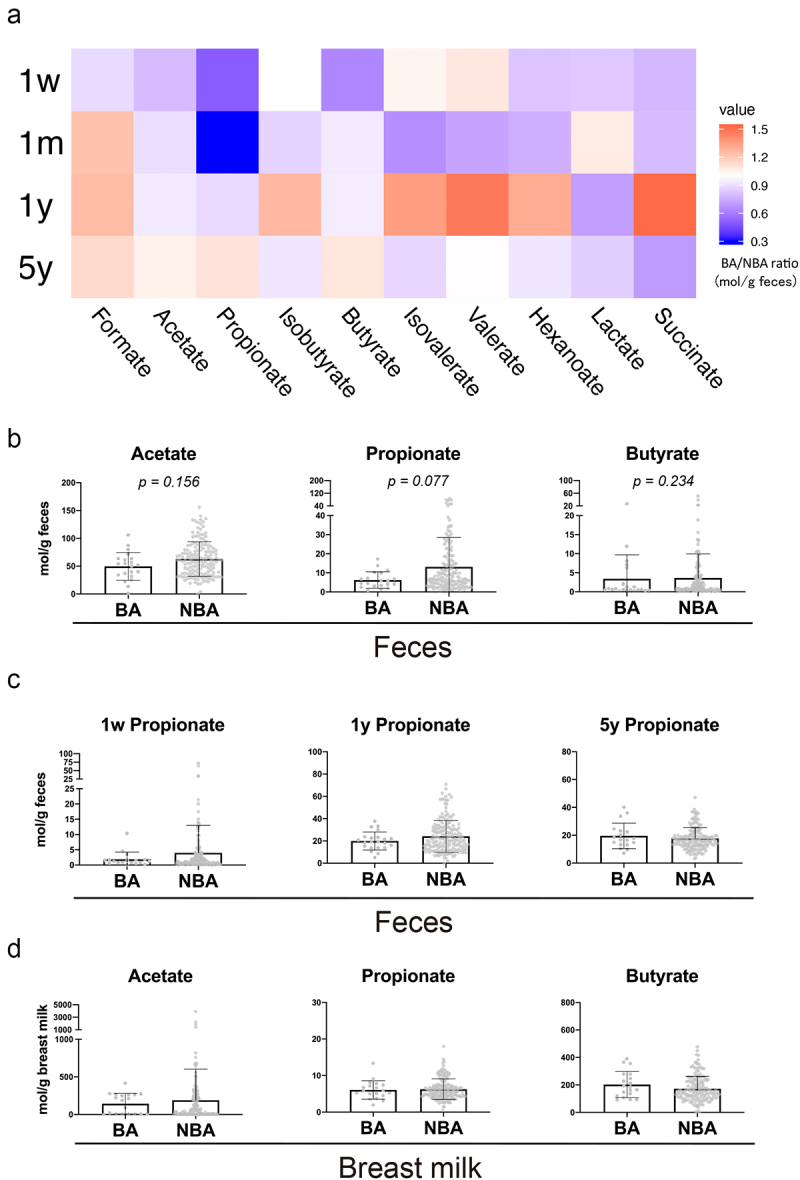
The concentration of fecal propionate was decreased in the human one-month-old BA group compared with the NBA group.Fecal (a-c) and breast milk (d) SCFAs are measured by gas chromatography (a) Heatmap of the ratio of the concentration (mol/g feces) of the ten most abundant fatty acids in feces of the BA group over that of NBA group at 1 week, 1 month, 1 year, and 5 years old. (b) Concentrations of the three most abundant SCFAs in the feces at 1-month-old. Data are mean ± SD. *P* values based on the Wilcoxon rank-sum test. (c) Concentrations of fecal propionate in the feces at 1 week, 1 year and 5 years old. Data are mean ± SD. *P* values based on the Wilcoxon rank-sum test. (d) Concentrations of the three most abundant SCFAs in the breast milk when the babies were 1 month old. Data are mean ± SD. *P* values based on the Wilcoxon rank-sum test.

We first performed a comprehensive gas chromatography-mass spectrometry-based targeted water-soluble metabolome analysis of fecal time course samples from the participants. Although statistically not significant, the BA/NBA ratio of fecal propionate at one month of age was lower among the SCFAs (Figure 5a,b), while this decrease was not observed at one week, one year and five years of age (Figure 5c). Moreover, our comprehensive analysis of water-soluble metabolites in addition to SCFAs revealed no significant differences between the BA and NBA groups (Supplementary Fig. S4A).

Since one report has noted a correlation between the concentration of SCFAs in the breast milk and the atopic phenotype^19^, it was possible that the fecal propionate in the BA group was derived from the breast milk; however, there were no significant differences in SCFA concentrations in the breast milk between BA and NBA groups at one week, one month, and six months (Figure 5d, Supplementary Fig. S4B, C). Taken together with previous studies reporting that fecal SCFAs are mainly produced by the fermentation and metabolism of indigestible dietary fibers by the intestinal microbiota^20–22^, these observations suggest that the difference in fecal propionate between BA and NBA groups is likely derived from the gut microbiota rather than from breast milk.

To assess bacterial taxa potentially producing SCFA independent of multivariate factors, the level of propionate obtained in a 1-month-old was modeled in the bacterial genus composition using a random forest model^23^. Interestingly, *Bacteroides* was the top hit for the propionate production score (Supplementary Fig. S4D). Moreover, we analyzed the correlation between fecal propionate concentration and the intestinal microbiota in the BA groups at one month of age. Three genera, *Varibaculum*, *Bifidobacterium*, and *Parabacteroides*, were positively correlated with fecal propionate in the BA group (Supplementary Fig. S4E). These results suggest that *Bacteroides, Varibaculum*, *Bifidobacterium*, and *Parabacteroides* may contribute to the production of fecal propionate, although further studies are required to test this hypothesis.

## Discussion

Here we report that, in the mouse model of HDM-induced allergic airway inflammation, the subsequent eosinophilic airway inflammation was attenuated and Th2 cytokine production was reduced when mice were given propionate during lactation. Moreover, we further discovered that these effects of propionate administration were mediated via GPR41 on eosinophils, likely through upregulation of TLR 2, 8 and/or 9. Finally, in our birth cohort, fecal propionate concentrations were reduced at one month in children who eventually suffered from asthma later in life. Taken together, our observations suggest that the propionate-GPR41 pathway during lactation has a protective role against the later development of HDM-induced asthma in a mouse model. This finding could be extended to human BA inflammation with possible therapeutic benefit.

Some studies in mice have indicated that changes in the gut microbiota affect the pathogenesis of allergic asthma^8,24,25^. Arrieta et. al. demonstrated that the colonization of germ-free mice with human fecal Lachnospira, Veillonella, Fecalibacterium, and Rothia improves ovalbumin-induced allergic airway inflammation^8^. However, these studies did not provide detailed mechanistic insight into how the gut microbiota impacts the pathogenesis of BA. Our study goes one step further and provides a hint into how the intestinal environment, including the gut microbiota, contributes to the regulation of BA, by showing that gut microbial-derived propionate can prevent allergic airway inflammation in mice.

The association between propionate and allergic airway inflammation has been reported by Trompette et al^26^, who showed in adult mice that propionate administration immediately protects from acute airway inflammation in a GPR41-dependent manner. By contrast, our present study suggests a new temporal aspect of the propionate effect in that intestinal propionate in the neonatal period can suppress the later onset of airway inflammation in adult mice, which could also be the case in the prevention of bronchial asthma in human children. Our findings also extend the study by Trompette et al. to show the importance of GPR41 expressed on eosinophils for the prevention of airway inflammation by propionate through TLR expression. In this study, we have added to the list of molecular mechanism of propionate, i.e., prevention of asthmatic airway inflammation. Interestingly, the cardioprotective effects of propionate are suggested to be mediated by Tregs^12^. Another study has also reported that propionate acts on monocyte-derived dendritic cells to reduce the release of several pro-inflammatory cytokines^27^. It is therefore possible that the airway protective effect of propionate observed in this study may also involve these anti-inflammatory functions; further studies will clarify the precise underlying mechanisms.

Some functions of SCFAs are transduced via GPCRs, GPR41 and GPR43, on the target cell surfaces. These GPCRs are abundantly expressed in various organs and cells and play a role in some chronic inflammatory diseases such as inflammatory bowel disease, obesity and arthritis^28–30^. Our present study indicates that propionate-mediated suppression of allergic airway inflammation is dependent on GPR41. GPR41 reportedly has both pro- and anti-inflammatory roles^30^, with some contradictory findings. Kim et. al. described that GPR41 promotes colitis via ERK and p38 deactivation in intestinal epithelial cells^31^. By contrast, Niranjana et al. have reported that GPR41^−/−^ mouse aortas exhibited a significant increase in vascular remodeling compared to wild-type mice^32^. Taken together, these observations may reflect pleiotropic roles of GPR41 in various diseases and a protective role in asthmatic airway inflammation.

We further showed that GPR41 is highly expressed on SILP eosinophils and that propionate upregulated their expression of some TLRs, probably via GPR41. The TLR family plays a guiding role in inducing innate immune responses to microbial pathogens^33^. Consistent with the fact that the TLR family has both pro-inflammatory and anti-inflammatory effects^33^, studies focusing on the TLR family in asthma have yielded contradictory results^34^. Our finding here suggests that the propionate signals through GPR41 play a protective role in BA via upregulating TLR signaling pathways in eosinophils. A previous study has reported that TLR agonists reduce lung eosinophilic infiltration during a virus infection in mice^35^, although it has not been clear whether the TLR agonists directly act on eosinophils. Taken together with our finding that the expression of some TLR genes in eosinophils was upregulated by propionate, a possible explanation is that the functions of eosinophils could be modulated by propionate via TLR signaling. Further studies are required to corroborate our finding of this protective role and to shed light on the underlying regulatory mechanisms of the propionate-GPR41-TLR axis in BA.

In our study, the human fecal samples were collected over a time period from one week after birth until five years of age. As with the microbiota composition, it is known that fecal SCFA concentrations undergo a drastic change within the first 100 days of life^36^. Moreover, the relationship between fecal SCFAs and BA has been noted in several large cohort studies of children^8,23^. For example, Depner *et al*. have also reported a consistent association between asthma phenotype and fecal butyrate levels, with a trend toward an association between atopic asthma and fecal propionate levels^23^. However, these studies are limited in that they have analyzed only one timepoint and have not captured the time course of SCFA concentrations in feces. By performing the repetitive analysis of fecal SCFAs over time, our study is novel in that we were able to detect changes in fecal SCFAs that may be associated with prevention of BA.

Although some bacterial genera have been reported to correlate with the development of childhood asthma^8,23^, few reports have directly analyzed the correlation between SCFAs in feces and intestinal bacterial composition. Our gut microbiota analysis showed a positive correlation between fecal propionate and *Varibaculum*, *Bifidobacterium* and *Parabacteroides*. Therefore, it could be possible that these bacteria are involved in the production of propionate in the neonatal intestine during the lactation period. Further studies are needed to clarify the point.

The suppressive effect of propionate was evaluated in an atopic asthma mouse model (house dust mite-induced allergic airway inflammation) in this study. BA includes another subtype, non-atopic asthma, although it is believed to be a minor component in BA clinical practice; the efficacy of propionate in this subtype should be studied in an appropriate model in the future.　Another issue to be resolved is that independent non-Japanese cohort studies have different results from ours^8,23^. These studies have reported significant differences in butyrate rather than propionate in pediatric bronchial asthma patients. This discrepancy could be due to differences in genotype, diet or gut microbial composition. Future analysis using a large human cohort may lead to a better understanding of the suppressive effect of propionate. Taken together, our study suggests that the propionate-GPR41-TLR axis in eosinophils during lactation may be involved in suppressing the development of BA. Although further studies are required, our results suggest that intestinal propionate could be a promising preventive target for the onset of BA.

## Materials and methods

### Mice

C57BL/6 mice (CLEA Japan, Inc.), GPR41-knockout mice on a C57BL/6 background^28^ and GPR43-knockout mice on a C57BL/6 background^37^ were housed in microisolator cages under specific pathogens-free (SPF) conditions in the animal facility of RIKEN Yokohama Campus. All experiments were performed as approved by the Animal Care and Use Committee of the RIKEN Yokohama Campus (Permission number: AEY2022–008). In some experiments measuring the fecal propionate concentration of weaning mice, mothers and offspring were housed in cages with a wire net flooring through which the fecal would pellets fall down, thus preventing coprophagy of the mothers’ feces by the offspring mice.

### SCFA administration

Pregnant wild-type C57BL/6 mice were given drinking water containing 200 mM acetate, 200 mM propionate, 200 mM butyrate, or control plain water, starting from E14 or right after delivery (noted in the Figures and their legends). For GPR41- and GPR43-knockout mice, pregnant mice were given drinking water containing 200 mM propionate or control plain water. Offspring were weaned at day 21 after birth and raised on plain water. Three weeks after weaning, the offspring were subjected to the HDM-induced allergic airway inflammation experiment described below.

### HDM-induced allergic airway inflammation

Mice were sensitized and challenged by intratracheal administration of HDM extracts (Greer Laboratories; XPB70D3A25) as described previously with minor modifications^38^. In brief, mice were sensitized intratracheally with 1 μg HDM in 50 μl PBS two days in a row. Seven days after the last sensitization, the mice were challenged with 10 μg HDM for four consecutive days. Seventy-two hours after the last HDM challenge, the mice were sacrificed and the numbers of eosinophils, neutrophils and lymphocytes in the BAL and lung were evaluated as described elsewhere^39^.

### Cytokine analysis by flow cytometry

Single-cell suspensions were prepared from the mLNs and BAL and stimulated with 20 ng/ml PMA (Calbiochem; 524400) and 1 μg/ml ionomycin (Calbiochem; 407950) for 4 h in the presence of 10 μM brefeldin A (BD Bioscience; 10 μM; 555029). Cells were stained with the indicated antibodies against cell surface molecules together with intracellular cytokines as described previously^40^.

## Histological analysis of the lung

Lung paraffin sections (5 μm thick) were stained with hematoxylin and eosin according to standard protocols. The histological score was evaluated as described elsewhere^41,42^.

### Elisa

The levels of HDM-specific IgG1 were evaluated as described previously^41^.

### qPCR analysis

qPCR was performed using a standard protocol on a Thermal Cycler Dice Real Time System TP800 (TaKaRa) using a TB Green Premix Ex Taq 2 Tli RNaseH Plus (TaKaRa; RR820A).

### RNA sequencing analysis

Total RNA was extracted from isolated intestinal eosinophils of propionate-fed and control mice by using the RNeasy Mini kit (QIAGEN; 74104). Library preparation was performed with a TruSeq RNA Library Prep Kit v2 (illumina; RS-122-2001). RNA sequencing was performed on an Illumina Nextseq 2000 in a 50-base single-end mode. mRNA profiles were calculated using R package feature count software and expressed as transcripts per kilobase million. Differentially expressed genes were determined by a weighted mean difference method using the IDEP.94^43^. GO biological analysis was performed with the Enricher^44–46^.

## Human cohort study design

This study was based on a cohort design to analyze the fecal microbiota of infants enrolled in the CHIBA Study^15^. This study includes 269 infants born at the Chiba University Hospital (Chiba, Japan) and Seikei-kai Chiba Medical Center (Chiba, Japan). The eligibility criteria of the study were the following: Japanese; infants of any sex, either of whose parents have any allergic disease; and who had obtained consent from a parent. We collected fecal samples at 1 week, 1 month, 1 year, and 5 years of age as much as possible, and mothers’ breast milk when the babies were 1 week, 1 month and 6 months of age. In the end, 65 subjects were lost during follow up periods due to hospital changes and consent withdrawal and the remaining 204 subjects were selected for metabolome analyses and 16S rRNA gene sequencing analysis on the one month fecal sample. Twenty-three of the 204 subjects later diagnosed with BA by a pediatric allergist according to the Japanese Guideline for Childhood Asthma 2014^47^ at 5 years of age were categorized as the BA group, while the rest were in the NBA group. All human experiments have been approved by the research ethics committee of the RIKEN Yokohama Campus and Chiba University (Permission number: H21–17(8)). Written informed consent was obtained from all participants.

## Sample collection and storage

The fecal and breast milk samples collected as above were immediately transported on ice to the laboratory. Upon arrival, the samples were immediately frozen in liquid nitrogen and stored at − 80°C until further analyses.

## Preparation of fecal samples

Aliquots (5 g) of feces were blended with 30 ml methanol and filtered with a 100 µm mesh filter to remove food residue after vigorous vortexing. The filtrate was centrifuged at 15,000 × g for 10 minutes at 4°C and the supernatant (methanol extract) was used for metabolomics analysis. Fecal microbiome DNA was extracted from the pellet.

## Extraction and measurement of SCFAs from breast milk and fecal samples

Extraction and measurement of SCFAs were as previously described^48^ with some modifications. 10 μl of Milli-Q water containing internal standards (2 mM [1,2-^13^C_2_] acetate, 2 mM [^2^H_7_]butyrate and 2 mM crotonate) were added to aliquots (90 μl) of breast milk. For fecal samples, aliquots (25 μl) of methanol extract were added to 10 μl of Milli-Q water containing internal standards and then centrifugally dehydrated at 40°C and reconstituted with 100 μl of Milli-Q water. Hydrochloric acid and 200 μl of diethyl ether were added to the solution and mixed well. After centrifugation at 3,000 × g for 10 min, 80 μl of the organic layer was transferred to a glass vial and 16 μl of N-tert-butyldimethylsilyl-N-trifluoroacetamid (Sigma-Aldrich; 394882) was added to derivatize the samples. The vials were incubated at 80°C for 20 min and allowed to stand at RT for 48 h before injection. The analysis was performed using a Shimadzu GCMS-TQ8030 triple quadrupole mass spectrometer with a capillary column (BPX5). The GC oven was programmed as follows: 60°C hold for 3 min, increased to 130°C (8°C/min), increased to 330°C (30°C/min) and finally a 330°C hold for 3 min. The detector and injector temperatures were 230°C and 250°C, respectively. GC was operated in constant linear velocity mode set to 40 cm/sec. Injection volume was set at 1 μl with a split ratio of 1:30. The data were processed and concentrations were calculated by LabSolutions Insight (Shimadzu).

## 16S microbial community analysis

Fecal DNA was extracted from 10 µg methanol-treated feces as described previously with minor modification^49^. The 16S rRNA gene analysis of DNA samples was performed as previously described^50^. Briefly, PCR was performed using 27 fmol 5-AGRGTTTGATYMTGGCTCAG-3′and 338 R 5-TGCTGCCTCCCGTAGGAGT-3′ to amplify the V1–V2 region of the 16S rRNA gene^50^. The amplified DNA samples (~330 bp) were subsequently purified using AMPure XP (Beckman Coulter; A63882), and quantified using a Quant-iT Picogreen dsDNA assay kit (Invitrogen; P7589) and a TBS-380 Mini-Fluorometer (Turner Biosystems). The 16S sequencing was performed using MiSeq according to the Illumina protocol. The paired-end reads were merged using the fastq-join program based on overlapping sequences. Reads with an average quality value of < 25 and inexact matches to both universal primers were filtered out. Filter-passed reads were used for further analysis after trimming off both primer sequences. For each sample, quality filter-passed reads were arranged in descending order according to the quality value and then clustered into OTUs with a 97% pairwise-identity cutoff using the UCLUST program version 5.2.32 (https://www.drive5.com). Taxonomic assignment of each OTU was made by similarity search against the Ribosomal Database Project (RDP) and the National Center for Biotechnology Information (NCBI) genome database using the GLSEARCH program^51^. Propionate score was calculated as described elsewhere^21^

## Data analysis

Data are summarized as mean ± SD, unless otherwise indicated. The statistical analysis of the results was performed using the Mann-Whitney U test, independent-samples t test or one-way ANOVA with post-hoc Tukey HSD Test as appropriate.

## Supplementary Material

Supplemental Material

## Data Availability

The 16S rRNA sequence data that support the findings of this study are openly available in DDBJ Sequence Read Archive at https://www.ddbj.nig.ac.jp/ddbj/index.html, reference number DRA014484. The RNA sequence dat that support the findings of this study are openly available in Gene Expression Omnibus at https://www.ncbi.nlm.nih.gov/geo/, reference number GSE207711.
