# Supplementary material for: The propionate-GPR41 axis in infancy protects from subsequent bronchial asthma onset
Source: Gut Microbes. 2023 May 2;15(1):2206507. doi: 10.1080/19490976.2023.2206507 (PMC10158560; doi:10.1080/19490976.2023.2206507)

Supplementary Figure 1

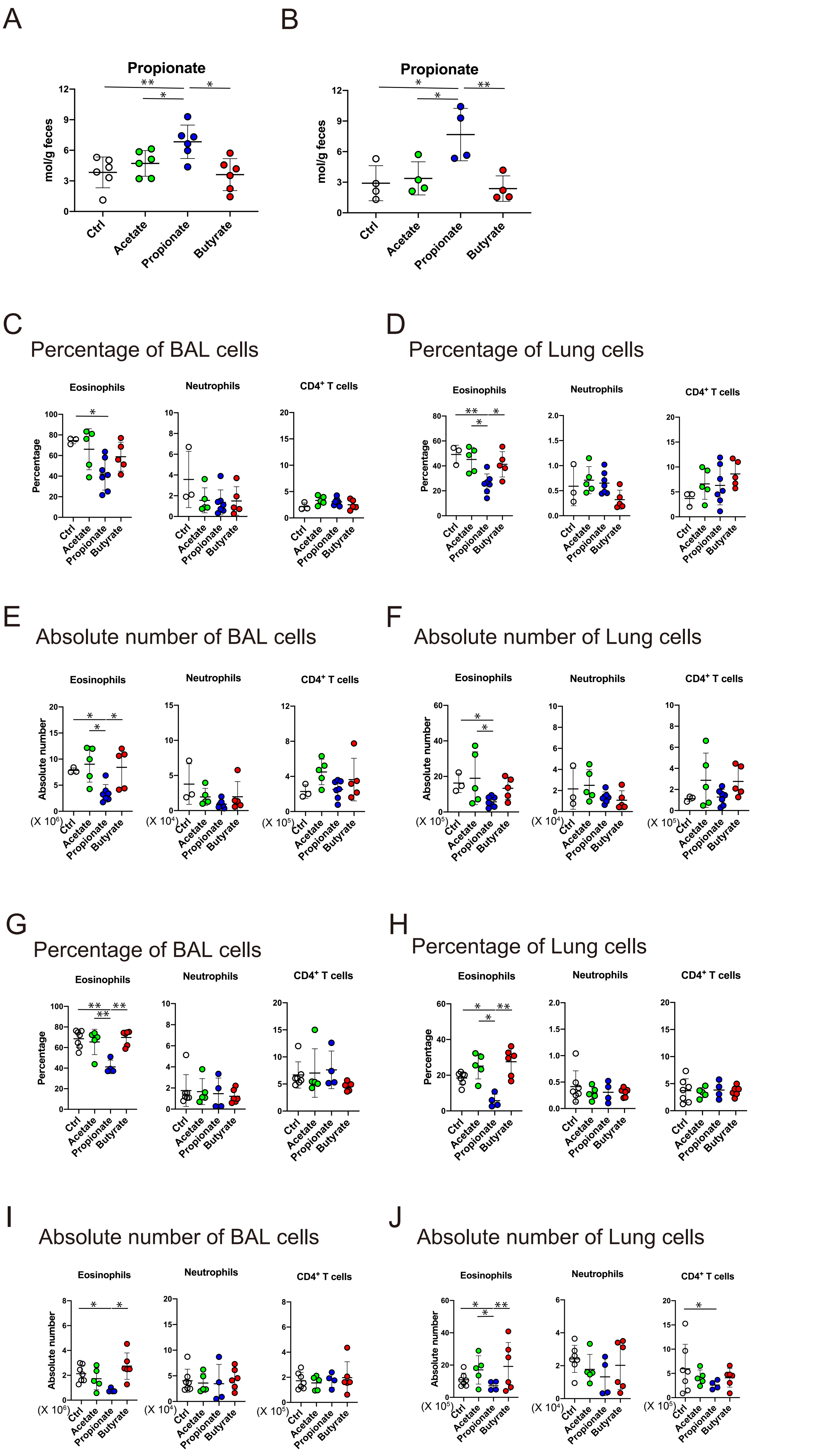

Supprementary Figure 2

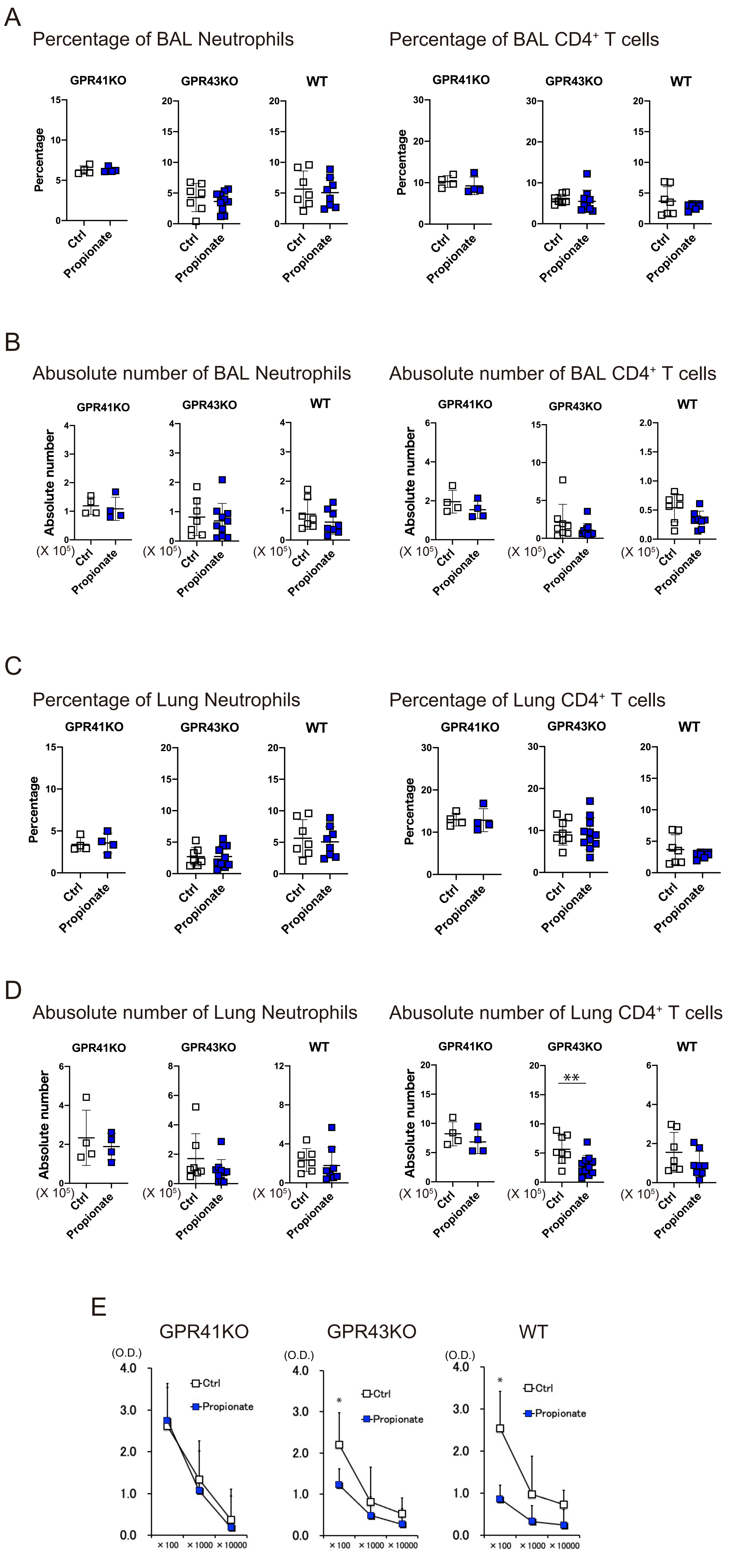

Supprementary Figure 3

A

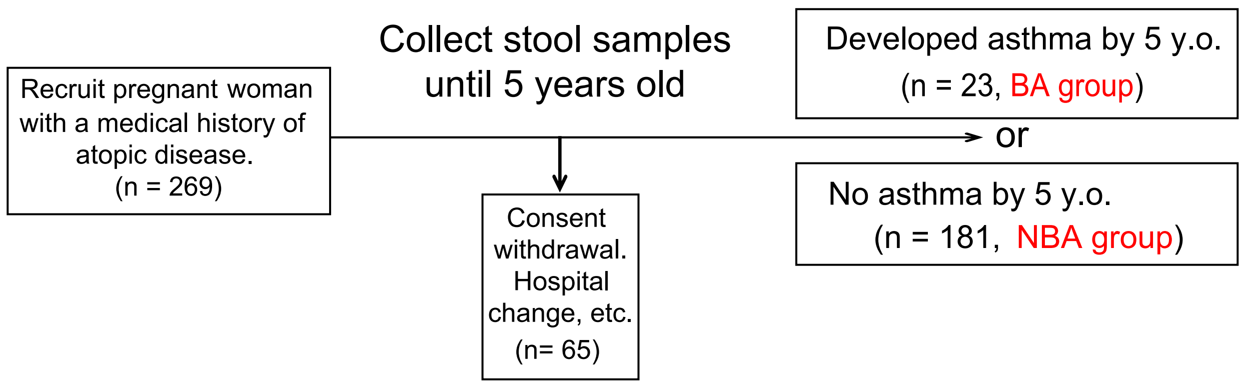

B

	BA group (n = 23)	NBA group (n = 181)	P value
Male (%)	87.0% (20)	47.0% (85)	<0.001*
Caesarean section (%)	17.4% (4)	32.6% (59)	0.137
Infant eczema (%)	21.7% (5)	31.5% (57)	0.338
Food allergy (%)	26.0% (6)	5.5% (10)	0.002*
Atopic dermatitis (%)	47.8% (11)	11.0% (20)	<0.001*
Hospitalized with pneumonia (%)	4.3% (1)	2.8% (5)	0.672
Birth weight (g)	2956.0	3032.0	0.473
Gestation period (Day)	272.2	275.1	0.345
Age of Mother (y.o.)	35.4	33.0	0.110
Nutrition form (Breast milk)	100% (23)	96.1% (171)	>0.999
Cat breeding (%)	4.3% (1)	5.5% (10)	>0.999
Dog breeding (%)	8.7% (2)	15.5% (28)	0.540
S. aureus skin colonization (%)	39.1% (9)	45.3% (82)	0.659
FGL mutation (%)	4.3% (1)	9.4% (16)	>0.999
History of Antibiotic use (%)	13.0% (3)	9.9% (18)	>0.999

Suppramentary Figure 4

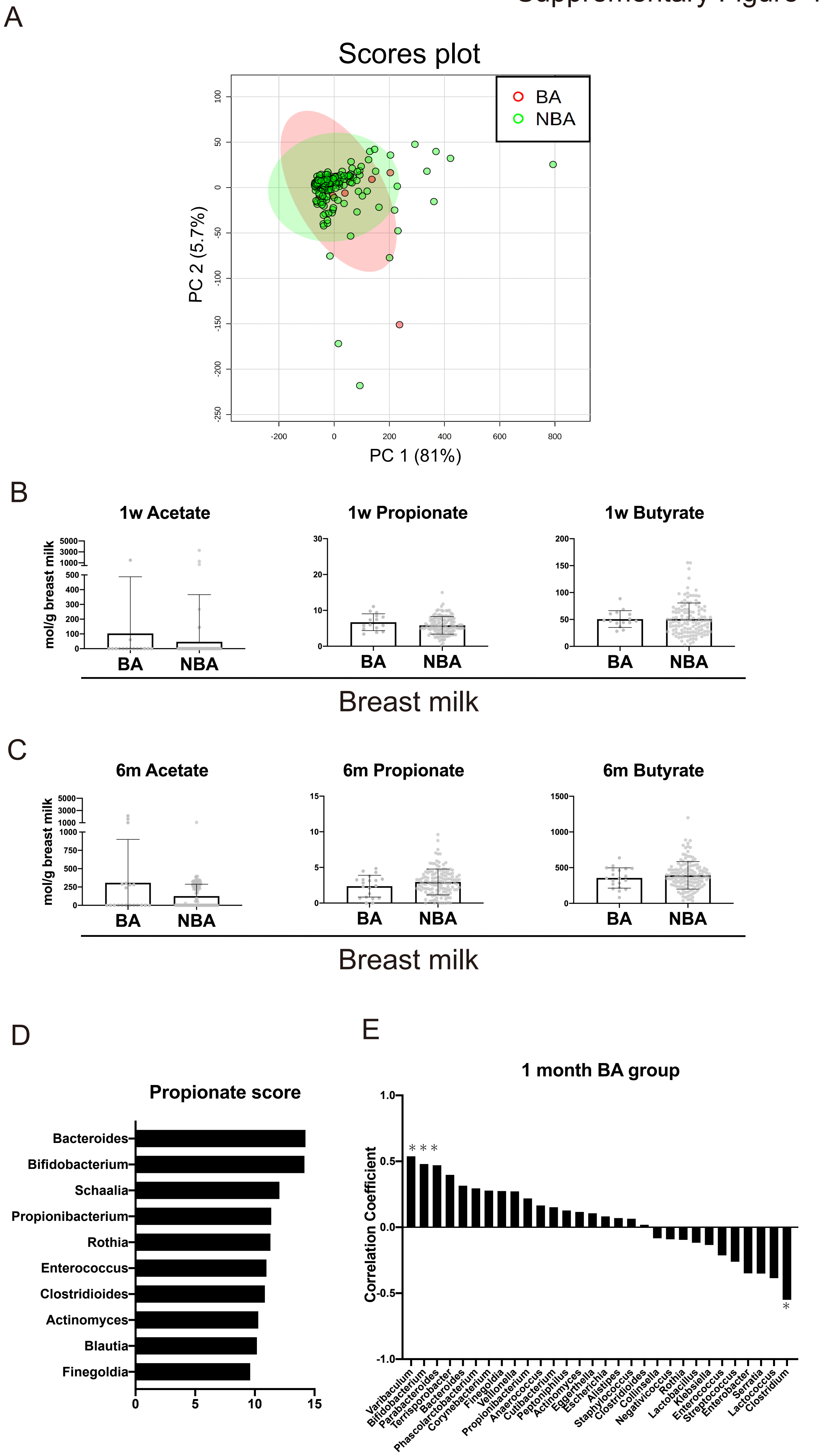

Supplement: Supplemental Material [file KGMI_A_2206507_SM5704.zip › Supplemental Figures/20230412_Fig-Supp.pdf]
