# Supplementary material for: The propionate-GPR41 axis in infancy protects from subsequent bronchial asthma onset
Source: Gut Microbes. 2023 May 2;15(1):2206507. doi: 10.1080/19490976.2023.2206507 (PMC10158560; doi:10.1080/19490976.2023.2206507)
Supplement: Supplemental Material [file KGMI_A_2206507_SM5704.zip › Supplemental Figures/Supplementary Figure legends.docx]

**Supplementary Figure 1: Propionate intake attenuates eosinophilic airway inflammation.**

**(A)** the level of propionate in the feces of 3-week-old offspring fed SCFAs and control water. Data are mean ± SD. *p < 0.05 and **p < 0.01 by one-way ANOVA and Tukey’s test. **(B)** the level of propionate in the feces of 3-week-old offspring housed in cages with wire net flooring where feces did not come in contact with the offspring mice. Data are mean ± SD. *p < 0.05 and **p < 0.01 by Student’s t-test. **(C-F)** The percentage **(C, D)** and absolute numbers **(E, F)** of inflammatory cells in the bronchoalveolar lavage **(C, E)** and the lung tissue **(D, F)** were evaluated in offspring mice whose mother mice were fed SCFAs or control water starting immediately after birth. Data are mean ± SD. *p < 0.05 and **p < 0.01 by one-way ANOVA and Tukey’s test. **(G-J)** The percentage **(G, H)** and absolute numbers **(I, J)** of inflammatory cells in the bronchoalveolar lavage **(G, I)** and the lung tissue **(H, J)** were evaluated 72 h after the last House Dust Mite challenge in male mice. Data are mean ± SD. *p < 0.05 and **p < 0.01 by one-way ANOVA and Tukey’s test.

**Supplementary Figure 2: GPR41 deficiency eliminates the inhibitory effect of oral propionate administration on HDM-specific antibody production.**

**(A-D)** The percentage **(A, C)** and absolute number **(B, D)** of neutrophils and CD4^+^ T cells in the bronchoalveolar lavage **(A, C)** and lung **(B, D)** were evaluated 72h after the last HDM challenge. Data are mean ± SD. *p < 0.05 and **p < 0.01 by Student’s t-test. **(E)** Serum levels of HDM-specific IgG1. Data are mean ± SD. *p < 0.05 by Student’s t-test.

**Supplementary Figure 3: Overview of the cohort study.**

**(A)** Summary of the participants in this cohort. **(B)** Background characteristics of the metadata of the BA and NBA groups. p values are based on the chi-square test.

**Supplementary Figure 4: The concentrations of breast milk SCFAs.**

**(A)** Principal Coordinates Analysis scores plots of water-soluble metabolites in the breast milk based on weighted and unweighted UniFrac distances between the BA group and the NBA group. **(B, C)** The concentration of acetate, propionate and butyrate in the breast milk at 1 week **(B)** and 6 months old **(C).** The SCFAs were measured by gas chromatography and normalized to weight. Data are mean ± SD. **(D)** Bar chart of predictive scores for propionate by random forest model. **(E)** Bar chart of correlation coefficients of fecal propionate with the relative abundance of fecal bacterial genus in 1-month-old babies in the BA group. *p < 0.05 by Spearman's rank correlation coefficient.
